# The impact of buckwheat and paulownia (*Paulownia elongata* × *P. fortunei*) intercropping on beekeeping value and buckwheat yield

**DOI:** 10.1038/s41598-024-72493-x

**Published:** 2024-09-14

**Authors:** Paweł Chorbiński, Marek Liszewski, Przemysław Bąbelewski, Anna Jama-Rodzeńska

**Affiliations:** 1 Department of Epizootiology with Exotic Animal and Bird Clinic, Wroclaw University of Environmental and Life Sciences, Grunwaldzki Square 45, 50-366 Wrocław, Poland; 2 Institute of Agroecology and Plant Production, Wroclaw University of Environmental and Life Sciences, 24A Grunwaldzki Square, 53-363 Wrocław, Poland; 3 Department of Horticulture, Wroclaw University of Environmental and Life Sciences, 24A Grunwaldzki Square, 53-363 Wrocław, Poland

**Keywords:** Agroforestry, Beekeeping value, Buckwheat, Nectar mass, Paulownia, Sugar mass, Plant sciences, Zoology, Climate sciences, Ecology, Agroecology, Biodiversity, Climate-change ecology, Ecosystem services, Forest ecology

## Abstract

Increasing crop diversity is a way for agriculture to transition towards a more sustainable and biodiversity-friendly system. Growing buckwheat intercropped with paulownia can contribute not only to mitigating climate change but can also enrich the environment with species of agricultural importance, without causing a decline in pollinators, since buckwheat is pollinated mainly by the honeybee. In a field experiment comparing growing buckwheat with paulownia against a monoculture crop, we investigated differences in flower visitation and beekeeping value, as well as the associated impact on crop yields. We analysed the effect of intercropping on the beekeeping value of buckwheat in terms of bee population size and the sugar mass in buckwheat flowers, nectar mass in buckwheat flowers, the quality of the delivered raw sugar and biometric characteristics. We found significant differences in the number of branches on the main shoot and the total number of branches. Significantly higher parameters were obtained in sites with buckwheat monoculture. The cultivation method variant did not cause differentiation in either the structure elements or the yield itself. Yields ranged from 0.39 (2021) to 1.59 (2023) t·ha^−1^. The average yield in intercropping was slightly lower (0.02 t·ha^−1^) than in the monoculture system of buckwheat (0.93 t·ha^−1^). More flowers per plant per day of observation and more flowers in millions of flowers per hectare per day of observation were observed in the intercropping of buckwheat with paulownia. Based on our experiment, we concluded that growing buckwheat in monoculture significantly increased the number of flowers, resulting in an increase in pollinator density and an increased number of pollinators per unit area.

## Introduction

Climate warming, global population growth and loss of biodiversity are urgent global issues that require action to transform agriculture for it to take a more sustainable direction^1–3^. The panacea for these changes has been seen in crop diversification and the fostering of agrobiodiversity^4–7^. Higher levels of crop diversity can be achieved through intercropping^8,9^. In intercropping, two or more plant species are grown on the same field at the same time. A particular form of intercropping is strip cropping (alley cropping)^10^, used instead of monocultures, which encourages the mass development of agrophages and leads to nutrient depletion and an increase in the abundance of certain weed species. The intercropping system allows the soil to be protected from erosion, excessive drying, enables pest reduction and reduces the presence of some weeds^11^.

Intercropping that involves melliferous plants protects pollinators, since the lack of floral resources in modern agricultural landscapes is considered to be a major factor driving pollinator decline^12,13^. Growing flowering plants in other plants' systems can be a way to improve farmland biodiversity^14,15^. Intercropping of trees as well as crop plants (like maize), and melliferous plants increases the biodiversity of the agroecosystem while allowing farms to generate additional income, promoting the development of biological life in the soil, reducing water losses, preventing nitrogen leakage from mineral fertilisers in the soil profile^11,16–18^.

The development of agroforestry systems under conditions that obtained in Poland seems particularly important in the context of mitigation of the negative effects of climate change, vast areas of marginal land, and a growing demand for bioenergy and organic produce. Strip-tree intercropping has the advantage of favourable shading, which indirectly contributes to increasing water retention, as paulownia trees have large leaves and the ability to store water. Inter-row shading by paulownia trees can protect the melliferous plants from too much sunlight exposure during the flowering period and thus prevent the decline in nectar secretion (desiccation of nectar). Nectar desiccation results in flowers becoming less attractive to pollinating insects, which leads to a decrease in seed yield^19,20^.

Three-quarters of our global crops depend in part on insect pollination necessary for yield, making pollinator management an essential part of their agricultural management^21^. Common buckwheat (*Fagopyrum esculentum* Moench) is a reliable and highly productive melliferous plant^22^. The large number of flowers that bloom over a long period contributes to making buckwheat fields a valuable and suitable site for bees^19^. Buckwheat honey is highly valued by professionals (nutritionists, doctors)—due to its specific organoleptic properties, such as its distinctive colour, pungent taste and molasses aroma—but is less favoured by consumers^23^. Regardless of the consumer's preferences, buckwheat honey has a very high nutritional value, paired with beneficial antioxidant and anti-inflammatory effects^24,25^. Buckwheat yield responds differently to an intercropping cultivation system. In a study by Lakhsmi et al. (2023)^26^ buckwheat intercropped with corn showed significantly higher plant height (90.4 cm) and dry matter production (5.33 g plant^-1^ ), followed by sweet corn (88.6 cm, 5.18 g plant^-1^). In contrast, Bardoloi (2020)^27^ found that better growth parameters, such as higher LAI which increased the photosynthetic area, increased light absorption, and thus greater dry matter accumulation, which leads to increased growth and yields in buckwheat alone compared to intercropping. The reduced growth of buckwheat in the intercropping was due to the effect of shade and competition for light between the main crop and buckwheat. Buckwheat is thus an important melliferous crop from a beekeeping point of view, which in favourable conditions can generate significant quantities of honey^28–31^. During its growth, over 2–3 months, a single buckwheat plant can develop 500–2000 flowers, each of which can potentially produce from 0.05 to 10 µl of nectar^31^. It has been found that a single foraging trip on buckwheat flowers can increase plant productivity by 25–30%^32^, three to four foraging trips are sufficient to pollinate one buckwheat flower, while a bee visiting more than five times reduces the productivity of the plant^32^.

Buckwheat nectar contains sugars, mainly hexoses (glucose and fructose), sucrose, vitamins and amino acids^33,34^. Concentration of sugars in the nectar is variable and depends on the time of day, ranging from 8 to 35%, though it may reach 51%^34–36^. Nectar production is sensitive to environmental stresses such as light deficiency, water stress and low temperature^36^. High temperatures and drought significantly reduce the amount of nectar produced by the plant, by up to 15 times, though the concentration of sugar in the nectar in such a situation increases.^36^ Despite the high concentration of sugars in the nectar, the attractiveness of buckwheat for pollinators under these conditions decreases, because the nectar dries out quickly in the nectaries, reducing its accessibility^35^. The average amount of honey raw material obtained from buckwheat ranges from 70 to 100 kg·ha^−1^ and for the best cultivars, can even amount to 150—300 kg·ha^−1^^29,37^^.^

According to Taylor and Obendorf (2001) ^38^ and Halbrecq et al. (2005)^39^ the position of flowers in a buckwheat cluster affects nectar production, which may be related to the supply of assimilates. Nectariferous paulownia flowers are a rich source of nutritious honey^40^. A flowering crop of *Paulownia tomentosa* allows to yield up to 700 kg of honey per hectare, and according to the manufacturer, the maximum yield of Oxytree is comparable^41,42^. In 2015, a new fast-growing tree was introduced into cultivation in Poland, under the commercial name Oxytree (Clon in Vitro 112). Paulownia Clon In Vitro 112 is a tree created in laboratory conditions by crossing and cloning two species: *Paulownia elongata* and *P. fortunei*. The Oxytree hybrid is characterised by rapid vegetative growth and is considered suitable for roundwood and biomass production and for revegetation. The yield obtained in the experiment of Chorbinski and Liszewski (2020)^43^ with Oxytree in Poland the potential availability of raw sugar for 1 hectare of 3-year crop of 237.5 kg of sugar yields about 300 kg of honey.

There is not much data regarding the evaluation of buckwheat intercropped with paulownia in terms of their beekeeping value. To the authors' knowledge, there have been nor such studies in Poland or worldwide. In formulation of our research hypothesis, we assumed that intercropping buckwheat and paulownia would result in favourable changes in the beekeeping value of buckwheat, and an increase in the number of visiting bees. The aim of the study was to determine the feasibility of growing paulownia and buckwheat (in the interrows between the trees) in the same field. Moreover, we investigated the beekeeping value of buckwheat grown in an alley system in terms of: the quantities of secreted nectar, sugar mass per 10 buckwheat flowers and per unit area (ha), as well as the potential raw sugar availability for bees.

## Methods

### Study system

A rigorous field experiment using the randomised block method including buckwheat (*Fagopyrum esculentum* Moench) and paulownia (Clon in Vitro 112—Oxytree; *P. elongata* × *P. fortunei*) has been set up in 2019 at the Research and Education Station of the Wrocław University of Environmental and Life Sciences (51°07′00″ N; 17°10 E, 121 above sea level). The factor under study was the intercropping of buckwheat between paulownia trees (AP). The experimental design included control sites (AK), i.e. buckwheat plots grown without paulownia. The buckwheat experiment ran from 2021 to 2023 and included five replicates. Oxytree seedlings were planted on 30.05.2019. In spring, on 19.05.2020, we carried out technical pruning to promote stem growth (future trunk). Buckwheat of the Kora variety was sown on 11.05.2021, 04.05.2022 and 20.04.2023 at a density of 250 seeds per square metre. The area of the buckwheat plot covered 30 square metres. The trees were planted in rows of five, the row spacing within the field was 5 m and the spacing between the trees in a row 4 m. The density of tree spacing was thus typical for Oxytree roundwood cultivation.

### Soil conditions

Our rigorous field experiment was based on a soil classified as humic ordinary alluvial soils (Type Ordinary alluvial soils—Polish Soil Classification (SGP6): SF), Subtype: humic ordinary alluvial soils—Polish Soil Classification (SGP6): SFh). The soil was classified as class F-IV b-a^44^. Soil samples for bioavailable forms of macronutrients (P, K, Mg) and mineral nitrogen analysis were procured prior to sowing buckwheat from several randomly selected locations within the field, separately for each site to obtain an average sample. The soil was sampled from the 0–30 cm layer using a soil sampler tool, air-dried, ground with a porcelain pestle and mortar, and sieved to < 2 mm. A portion of each sample was then finely ground for analysis. Soil testing included the determination of basic soil characteristics and properties, such as soil morphology, systematic position, use value, pH, macronutrient content, including mineral nitrogen content. We carried out the characterisationof the following soil properties:

- content of bioavailable forms of P and K—using the Egner-Riehm method; where phosphorus and potassium compounds in a soil were extracted with a lactate buffer consisting of calcium lactate and lactic acid. The extraction solution used had pH  3.55 (at this level of acidity, extraction conditions are maintained regardless of the initial soil reaction); soil to solution ratio: 1:50, time of extraction 90 min;
- available magnesium content—using Schachtschabel method with 0.0125 M calcium chloride as extraction solution; the ratio of soil to extraction solution is 1/10 (m/v), using the ratio of 5 g air-dry weight of soil sieved through a 1 mm sieve and 50 cm^3^ extraction solution. The extraction time in a rotary stirrer is 120 min, at 40 revolutions per minute
- pH in distilled water and in 1 M KCl—using potentiometric pH metres, Soil pH was measured with a glass pH electrode (1:5 soil:deionized water, measurements after 30 min)
- nitrogen content—using the modified Kjehdal method (total nitrogen determination) which consists of several stages: combustion of the organic matter with concentrated sulfuric acid; release from ammonium sulfate , ammonia by distillation from an alkaline environment; quantitative binding of distilled ammonia to ammonium sulfate.

The soil pH in both the intercropped site and the control site was slightly acidic both before the experiment had been set up and at the end of the study period. Phosphorus and potassium contents on both sites were very high in the year the experiment started and at the end of the study period—phosphorus level was high (AK) and very high (AP). Towards the end of the 2023 season, the potassium content was found to be reduced to ‘high’ (AK). The magnesium content was very high in the soil in both sites in the year the experiment started, while by the end of 2023, it had decreased to medium (Table 1). Over time, the mineral nitrogen content in the soil decreased.

**Table 1:** Soil pH and macro-element content of the soil profile (0–30 cm) in 2019 and 2023.

Detailed determination	2019		2023	
	AK	AP	AK	AP
pH in KCl (soil pH)	6.2 (slightly acidic)	5.8 (slightly acidic)	5.8 (slightly acidic)	5.9 (slightly acidic)
Phosphorus P_2_O_5_ (mg/100 g of soil)	48.5 (very high)	27.6 (very high)	18.7 (high)	22.9 (very high)
Potassium K_2_O (mg/100 g gleby)	33.5 (very high)	33.5 (very high)	22.3 (high)	25.9 (very high)
Magnesium Mg (mg/100 g gleby)	7.6 (very high)	7.6 (very high)	6.7 (medium)	5.4 (medium)
N min. (kg/ha)	43.8 (very low)	48.0 (very low)	56.8 (low)	21.4 (very low)

AP—intercropping, AK – control, N min. – mineral nitrogen.

### Weather conditions in 2021–2023

We used Selyaninov's hydrothermal coefficient (HTC) to describe the impact of weather conditions on the buckwheat and paulownia development, using the following formula:

$${\text{K }} = {\text{ P }}/ \, \left( {0.{1} \cdot {\text{T}}} \right)$$

where: K is the Selyaninov's hydrothermal coefficient, P is the total rainfall in the months IV–X, T is the sum of daily average temperatures in specific months^45^.

The 2021 season should be considered as exceptionally dry, with 45% less rainfall in the months IV–X, compared to the multi-year period (Table 2). The low Selyaninov's hydrothermal coefficient (HTC) for the months VI-X indicate soil drought or drought. In June and September, high temperatures, well above the multi-year averages for these months, exacerbated soil water deficits. In contrast, the relatively low temperatures of April and May were responsible for the delayed resumption of paulownia’s growth.

**Table 2:** Weather conditions and Sielianinov's hydrothermal coefficient in 2021–2023 by meteorological observation station (AsterMet) in Swojec near Wroclaw.

Month	Temperature (°C)				Rainfall (mm)				HTC (K)		
	2021	2022	2023	*Mean* 1990 – 2020	2021	2022	2023	*Mean* 1990 – 2020	2021	2022	2023
IV	7.5	6.4	8.1	**9.6**	32.5	32.7	31.8	**32.8**	1.45	1.46	1.31
V	12.4	12.7	13.2	**14.3**	59.0	20.9	23.9	**58.9**	1.54	0.44	0.60
VI	19.8	20.1	18.7	**17.8**	38.4	39.7	73.4	**74.6**	0.65	0.58	2.09
VII	20.5	20.7	20.7	**19.7**	41.1	132.5	50.5	**86.6**	0.64	2.12	0.79
VIII	17.3	17.7	19.9	**19.2**	11.0	92.5	144.1	**63.6**	0.21	1.45	2.33
IX	15.0	14.8	17.0	**14.2**	14.5	82.0	4.7	**50.6**	0.32	2.12	0.92
X	9.5	9.1	12.0	**9.3**	10.2	8.6	30.0	**40.8**	0.35	0.25	0.81
Mean/sum (IV-X)	14.6	14.5	15.7	**14.5**	206.7	408.9	358.4	**377.0**	–	–	–

Significant values are in bold.

In the 2022 season, the distribution of precipitation across the months was uneven. Low Selyaninov's hydrothermal coefficients (HTC) for May and June indicate soil water deficit. There was a significant improvement in July, with rainfall exceeding the average multi-year total by 53%. The following summer months also had rainfall exceeding the average multi-year totals for the months of VIII and IX. In June and July, average monthly temperatures significantly exceeded multi-year averages by 2.3 and 1.0 °C, respectively. In the 2022 season, total precipitation in the IV-X period exceeded the multi-year average for that period (by 8.5%), while the average temperature did not differ from the mean.

In 2023, buckwheat emergence was prolonged due to low temperatures, while paulownia trees also resumed their growth late. Spring in the 2023 season was marked by low temperatures and low rainfall in May. The average temperatures of April and May were lower than the multi-year averages for these months by, respectively: 1.5 and 1.1 °C. In the following months, we recorded temperatures above the multi-year averages, with a precipitation deficit in the months of May, July, September and October. August was the only month when we recorded very abundant rainfall, exceeding the multi-year average total by as much as 127%. The growing season for paulownia lasted from 163 days (in 2023) to 192 days (in 2022) (Table 3).

**Table 3:** Selected morphological and flowering-related traits of buckwheat (averages for combination and individual years).

Specification	Plant height	Number of branches on the main shoot	Total number of branches	Number of inflorescences per plant	Total number of seeds
AP (intercropping)	49.2	0.98	2.51	5.39	26.4
AK (control)	49.6	1.36	3.04	6.13	26,4
LSD_0.05_	**ns**	**0.37**	**0.43**	**ns**	**ns**
2021	47.3	1.41	2.36	6.84	15.5
2022	47.1	1.38	4.22	5.55	28.8
2023	53.9	0.72	1.74	4.89	34.9
LSD_0.05_	**5.27**	**0.45**	**0.53**	**1.33**	**8.5**

Significant values are in bold.

### Growing conditions/agro-technology

Prior to setting up the experiment, following the 2018 winter triticale harvest, the field was ploughed with a subsoiler plough and then the soil was kept weed-free using a cultivating unit (rotary harrow with a crushing roller). In autumn, deep pre-winter ploughing was carried out to a depth of 25 cm. In the year when the experiment started, i.e. in the spring of 2019, the field was ploughed once again with a subsoiler plough, and the soil was further amended using a cultivating unit. Following paulownia seedlings' planting, the soil in the inter-rows was loosened using a rotary harrow. The following year, in the spring of 2020, the soil was ploughed shallow and then fine-tilled using a cultivator. After technical pruning of the young trees in May of that year, the inter-rows were tended mechanically. In 2021, after winter, we carried out shallow ploughing using a subsoiler plough, followed by a cultivating unit equipped with a string roller. By May of that year, harrowing was performed twice to control weeds. Following the buckwheat sowing, the paths were tended mechanically several times, until the crop was harvested using a combine harvester. Before winter, the soil was kept weed-free by harrowing using a rotary harrow. Tillage in 2022 included harrowing the field after winter using a cultivator, followed by ploughing with a subsoiler plough to a depth of 12 cm. Before buckwheat sowing, the soil was treated using a cultivating unit consisting of a rotary harrow and a crushing roller. The sowing was done using a field seeder. The paths between the plots were tended during the growth period.

Similarly, post-winter tillage in 2023 included shallow ploughing with a subsoiler plough, followed by a cultivator with a string roller before sowing buckwheat. After the buckwheat was sowed, paths between strips were being cultivates until the buckwheat was harvested using a plot combine harvester.

No mineral or organic fertilisers or pesticides were used in the experiment.

### Illumination in the buckwheat field

In 2023, the period of full flowering of buckwheat (when flowers were picked to determine nectar secretion), we measured illumination at the level of plants' upper leaves in each plot, on five dates. Measurements were made under different cloud coverage conditions, between 8.30 a.m. and 12.30 p.m. In plots of buckwheat and paulownia intercropping (AP), illumination was recorded in the shade of the tree crowns. All measurements were made using a Czech-made PU-550 light intensity meter.

### Analysis of buckwheat and paulownia biometric traits and the yield

Before harvesting the buckwheat, we randomly sampled 10 plants from each plot. The samples were sourced from the middle rows to mitigate the border effect. The biometric analysis included: plant height, number of branches, number of twigs, number of inflorescences, number of seeds and weight of full seeds per plant. The yield per plot was adjusted to 15% moisture content and converted to tonnes per hectare.

For paulownia, we measured tree height, stem girth at breast height (measured at 130 cm) and crown dimensions.

### Pollinator management

Nectar secretion from buckwheat was determined using the pipette method developed by Jabłoński (2002)^46^ on eight dates in 2021 (between June 28–July 14), eleven dates in 2022 (between June 13–July 14) and ten dates in 2023 (between June 7–June 28). Flower samples (from at least 10 plants, from at least 10 plants protected from insect visits) were collected from the center of the canopy of each plot. The collected nectar was weighed in a laboratory and the concentrations of sugars in it were measured using an Abbe’s refractometer (KERN ABBE Refractometer ORT 1RS). The sugar mass was calculated using the formula: sugar mass = (nectar mass × % concentration of sugars)/100. The result was then converted to show value per 10 buckwheat flowers. At each date, the number of developed flowers on 10 plants was also counted and converted to account for plant density per 1 m^2^ and per hectare. In this way, we could calculate the availability of raw sugar for bees per 1 ha/day of measurement. For each day of observation, we also determined the number of bees visiting the plot (block) between 7.30 a.m. and 8.00 a.m.

### Statistical analysis

To assess the impact of the cultivation method across different years of study on the biometric traits and yield of buckwheat, we performed a two-factor ANOVA analysis using Statistica 13.1 software. In case of significant differences between means, we compared them using the HSD-Tukey test, with a significance level of α = 0.05. We included mean results, maximum, minimum values as well as standard deviations. Shapiro test normality was performed for evaluation of nectar secretion as well as one-way Anova analysis. All analyses were conducted at the 5% significance level using the STATISTICA (data analysis software system) package, v. 13.1 by StatSoft, Inc. (2017)^47^. To assess the differences between the mean values of the following parameters: mass nectar of 10 flowers of buckwheat (mg), mass sugars of 10 flowers of buckwheat (mg), average number of flowers per 1 plant per 1 day of buckwheat, average number of flowers (million/1 ha^−1^ per day of observation, availability of raw sugar (kg per 1 ha), average number of bees for 1 block (pcs) between groups (AP) and (AK) we applied Student's independent samples *t* test. We performed separate analyses for each year in the 2021–2023 period and a joint analysis for all samples collected over three years. The figures were prepared with Statistica version 13.3.

## Results

### Buckwheat and paulownia development phases

The start of the vegetation phase of both intercropped species occurred in early May, regardless of the year. Buckwheat emergence in the 2022 season was half as long as in the previous year, while in 2023 extended over as many as 18 days, when it was prolonged due to low temperatures. The vegetation phase for paulownia trees also resumed late in 2023. Buckwheat developed flower buds at between 30 days (2022) to 41 days (2023) following sowing. Full flowering lasted from 18 days (2022) to 33 days (2021). The shortened flowering period, especially in 2022, was due to high air temperatures in the months VI–VII and the absence of rainfall during this time. During the 2023 season, full flowering lasted 29 days. Buckwheat was harvested after the following periods: 85 days (2021), 69 days (2022) and 117 days (2023) after sowing.

### Effect of intercropping on biometric traits, flowering biology and yield of buckwheat

The cultivation system had no significant impact on the variation in the morphological traits of buckwheat, except for the number of branches on the main shoot and the total number of branches (Table 3), which may have been due to increased sun exposition in the control variant, where buckwheat branched more strongly. These traits were mainly modified in line with the weather pattern in the specific years of the study. The total number of set seeds was significantly lower in the 2021 season when compared to other seasons. The tallest plants, which were also the least branching, occurred in the 2023 season.

**Fig. 1:**
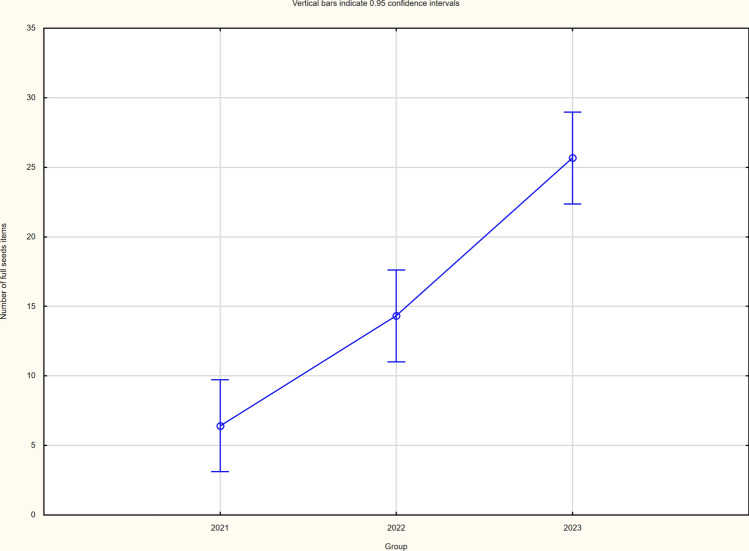
The number of full seeds (average for each year for each of the study).

**Fig. 2:**
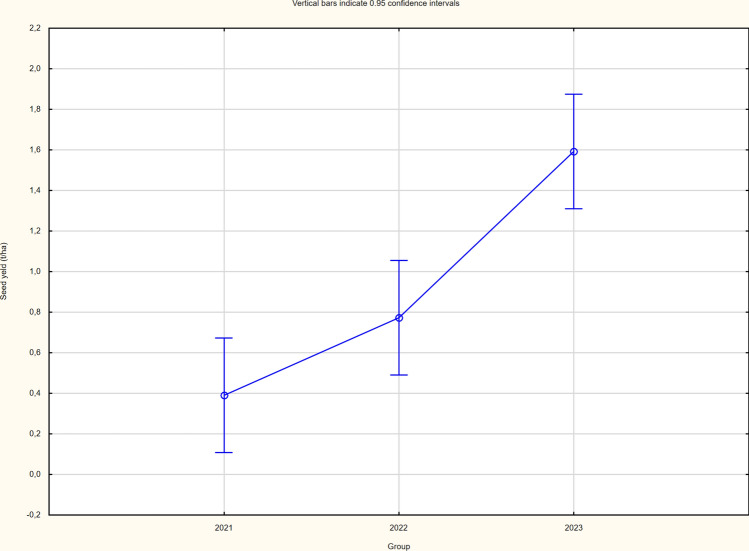
Seed yield of buckwheat cornels in (average for each year of the study).

**Fig. 3:**
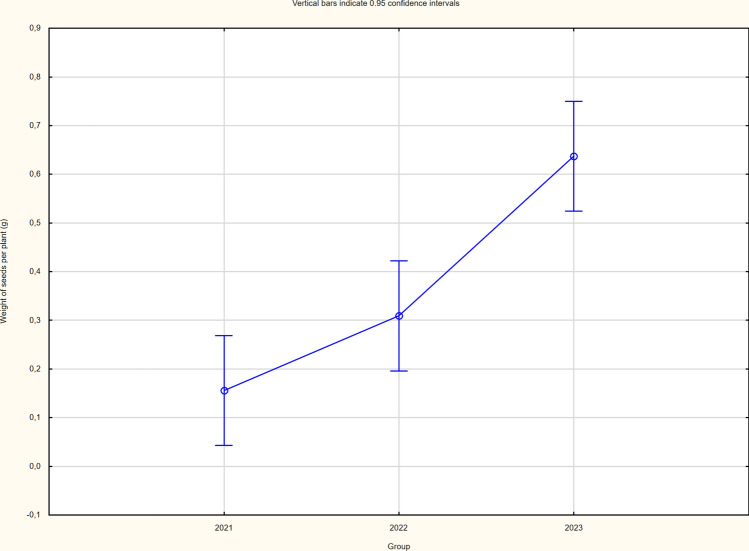
Weight of seeds per plant of buckwheat (average for each year of the study).

The cultivation method variant did not cause differentiation in either the structure elements or the yield itself (Table 4). There were significant differences in yield in individual years of the study, with the lowest seed yield recorded in 2021 due to the least favourable weather conditions and the highest in 2023 (Figs. 1, 2 and 3). The significantly higher yield of buckwheat in the 2023 season was influenced by the significantly higher number of whole seeds and seed weight per plant. Yields ranged from 0.39 (2021) to 1.59 (2023) tonnes per hectare. The yields of buckwheat between the two cultivation system did not vary significantly (Table 4).

**Table 4:** Seed yield and its structure (average for combinations).

Specification	Number of full seeds items	Weight of seeds per plant (g)	Seed yield (t/ha)
AP (intercropping)	16.1	0.36	0.91
AK (control)	14.9	0.37	0.93
LSD_0.05_	**ns**	**ns**	**ns**

Significant values are in bold.

### Selected trunk and crown parameters of paulownia in 2021–2023

The average height of the trees at the beginning of the growing season 2021 was 160.5 cm, at the end of the 2022 season 311 cm, and in 2023—507 cm. Tree crown sizes measured in October 2022 were on average: 156 (height) × 209 cm (width), while in 2023, 308 (height) × 357 cm (width). Trunk girths at breast height (measured at 130 cm) at the end of the 2021, 2022 and 2023 seasons were respectively: 16.4 cm, 17.9 cm, 22.7 cm. No flowering of paulownia was recorded during the years covered by the study.

### Effect of intercropping on illumination during the 2023 season

Illumination varied between the sites under study (Table 5). It was found to be higher in the buckwheat monoculture crop, suggesting that paulownia may reduce light for buckwheat. At the same time, a lower solar exposure may have reduced the effects of soil drought stress, as the trees have contributed to increased water retention. It should be noted that during the early period of buckwheat development (emergence and foliage), paulownia and buckwheat are, to a degree, in competition for light.

**Table 5:** Illumination during buckwheat bloom in lux (lx).

Data	Measurement		Comments
	AP	AK	
05.06.2023	19.2	75.3	Cloudless, sunny, warm, buckwheat budding, 10.00 a.m
LSD_0_._05_	16.0		
07.06.2023	31.3	91.1	Clouds/sunshine, "subdued" sky, dry, warm, first flowers, 12.30 p.m
LSD_0.05_	21.6		
12.06.2023	9.3	58.5	Sunny, dry, warm, full bloom, 9.00 a.m
LSD_0.05_	10.2		
14.06.2023	19.4	28.1	Cloudy, full flowering, before storm, 11.00 a.m
LSD_0.05_	3.4		
26.06.2023	12.1	57.8	Sunny, very warm, first brown seeds, 8.30 a.m
LSD_0.05_	20.3		

### Effect of intercropping on the beekeeping value of buckwheat

The results show a clear influence of weather on the relevant parameters. The mass of nectar and sugars per 10 buckwheat flowers reached the highest values in 2023 for both groups. The lowest values for these parameters were not as consistent, with 2022 for the AP group and 2021 for the AK group. The established availability of raw sugar for pollinators demonstrated that 2022 was the least favourable year, with the lowest values for both groups. It was interesting to note that in 2023 buckwheat had the lowest number of flowers per plant and per unit area, but due to the high sugar content in nectar and nectar mass, the AK group achieved the highest result. The AP group had a lower availability of raw sugar in this period, but the difference was not statistically significant (Table 6). Assessing the averages for the three-year period, we found that the mass of sugars per 10 buckwheat flowers in the AK group was slightly higher (0.55 mg) than in the AP group. We found a similar relationship when assessing the nectar mass per 10 flowers, with higher values in the control group. During the study period, buckwheat in the AP group showed the higher number of developed flowers than in the control group, which was reflected in the number of flowers per hectare per observation day. However, when converted into raw sugar availability, the increased number of flowers in the AP group did not push the value in this parameter beyond that in the AK control group (1.95 kg and 2.12 kg, respectively). For both groups, there was also no change in the attractiveness of the crop to bees, and the values obtained were not markedly different (Table 6). The statistical analysis did not show the significance of differences between the two groups in the three-year period for the buckwheat parameters presented below. The observed differences between years are mainly due to the impact of weather during the buckwheat growing season. 2023 proved to be the most favourable year, while 2022 was the least favourable. As we found no significant differences between the control group and the group assessed at the 5% level, we did not proceed with further statistical analyses.

**Table 6:** Beekeeping value of buckwheat in intercropping.

Specification	Parameter	2021		2022		2023		Years 2021–2023	
		AP	AK	AP	AK	AP	AK	AP	AK
Mass of sugars of 10 flowers of buckwheat (mg)	x̅	**0.46**	**0.42**	**0.38**	**0.46**	**0.64**	**0.75**	**0.49**	**0.55**
	SD	0.09	0.12	0.12	0.11	0.21	0.27	0.18	0.23
	*P* value	0.710		0.377		0.069		0.208	
Mass of nectar of 10 flowers of buckwheat (mg)	x̅	**4.18**	**3.78**	**3.79**	**4.92**	**6.10**	**7.83**	**4.69**	**5.61**
	SD	1.99	2.24	2.81	3.03	2.03	2.00	2.49	2.96
	*P* value	0.439		0.126		0.345		0.334	
Average number of flowers on 1 plant per 1 day of observation	x̅	**18.56**	**21.08**	**18.14**	**14.47**	**12.99**	**13.38**	**16.48**	**15.92**
	SD	3.37	6.11	6.75	4.72	4.45	3.14	5.67	5.58
	*P* value	0.410		0.155		0.822		0.713	
The average number of flowers million/1 ha^−1^ per day of observation	x̅	**46.40**	**52.70**	**45.35**	**36.18**	**32.48**	**33.45**	**41.20**	**39.80**
	SD	8.44	15.28	16.87	11.79	11.11	7.85	14.17	13.96
	*P* value	0.325		0.155		0.822		0.705	
Availability of raw sugar in kg per 1 ha	x̅	**2.14**	**2.25**	**1.72**	**1.65**	**2.05**	**2.53**	**1.95**	**2.12**
	SD	0.62	1.19	0.73	0.64	0.92	1.20	0.77	1.05
	*P* value	0.825		0.677		0.322		0.566	
Average number of bees for 1 block (pcs.)	x̅	**20.93**	**30.30**	**10.89**	**8.85**	**37.80**	**32.10**	**22.94**	**22.79**
	SD	7.64	8.46	12.93	11.50	40.73	38.61	27.29	25.84
	*P* value	**0.036**		0.700		0.752		0.983	

Significant values are in bold.

When assessing the number of bees visiting the buckwheat plots, we noted no differences in the three study years, but in 2021 there was a statistically significant increase in bee visits in the AK group compared to the AP group (*p* = 0.036)*.*

## Discussion

Intercropping is a system in which two or more crop species are sown or planted simultaneously in the same field and during the same growing season^48,49^. The benefits of such cultivation include reduction of the impact of biotic and abiotic environmental stresses on yield—above all, the protection of the soil against erosion and excessive evaporation of water from the soil. When planning intercropping of several plant species, one should consider possible adverse effects of allelopathic plant activity, light distribution between crops, root system structure, development and growth rate, while maintaining botanical distinctiveness^50–52^.

The primary factor determining high yield is light. Several studies have found that competition for light is greater than the competition for soil resources in temperate and humid climate regions, where crops grown in such a system are usually fertilised and rainfall is relatively abundant, providing an adequate supply of water^51,53^. Bouttier et al. (2014)^50^ demonstrated that the availability of light for intercropping near hybrid poplar was lowest due to tree size and planting density per row. Reduction in crop yield, high ratio of root density per unit of intercrop biomass, and high values of hybrid poplar (*Populus deltoides, Populus nigra*) root densities per area, as well as low soil nitrogen concentration all suggest depletion of soil resources. The impact of poplar in the study by Alam et al. (2014)^54^ on agricultural crop yield was greater than for red oak. In the study by Bouttier et al. (2014)^50^ the yield of plants (*Phleum pratens* L. and *Trifolium pratense* L.) decreased near trees, especially near hybrid poplars. This was mainly due to competition for light and roots competition for resources, even if soil resources and fertilisation levels were low. In our study, there was no significant effect of intercropping on buckwheat yield, either in terms of competition for light or for soil resources.

In intercropping involving well-matched species, interspecies competition proves stimulating for both cultivated species or at least one of them, which manifests in an increase in yield quantity and quality. Changes in soil properties in an orchard where intercropping was used had a positive effect on walnut productivity. Walnut is a deciduous species with late developing leaves, so shading by its crown, which coincides with later stages of barley development, may not have a critical limiting effect on barley yield. In addition, barley is a C3 crop, which means it is less susceptible to the negative impact of shading, as only 50% of full sunlight is sufficient for the plant to become fully light-saturated^55^. Intercropping with winter barley was 53% more productive per unit area and 83% more water-intensive than growing walnut and barley separately, but also 48% more productive per unit area and 70% more water-intensive than the walnut-barley system^52^.

Yield advantage in intercropping is measured by the so-called competition functions, such as relative yield total (RYT), relative value total (RVT) or the yield equivalent to the base-level yield. In an experiment by Mandal et al. (2014)^55^ recorded 5.48 t ha^−1^ of maize equivalent yield (MEY) in maize and soya intercropping (1:2) against 2.48 t ha^−1^ for just maize. The relative yield total (RYT) of the intercropping was higher than in monoculture in the different experiments, indicating a yield advantage^56^. Manasa et al.^57^ mentioned that MEY was 7.6 t ha^−1^, when maize was intercropped with groundnut (2:2), compared to a maize yield in monoculture, which was 5.7 t ha^−1^, while RYT amounted to 1.47. In our study, the average yield of buckwheat in monoculture was 0.93 t ha^−1^ and in intercropping was 0.91 t ha^−1^ and the difference was not statistically significant.

The value of crop yield in intercropping is linked to the compensation principle. The probability of yield reduction due to disease, pests or extreme weather conditions is significantly lower in intercropping. It has been shown that at any level of stress factor, intercropping has a significantly lower probability of failure than monoculture^58^. In intercropping, one species can change the microclimate for the other species, which can keep the emergence of pests and diseases in check, resulting in greater productivity and stability^59^. Intercropping tends to be more popular in developing countries, perhaps because it is assumed that it gives more stable yields than monocultures^60^. Many field experiments have looked into yield stability in intercropping and the results are sometimes inconclusive^61–63^. Li et al. (2020)^48^ noted that an absolute yield increase in intercropping, compared to monoculture was greatest for maize intercropped with short-grain cereals or legumes.

Alekseyeva and Bureyk (2000)^36^ demonstrated that nectar productivity depends on the flowering stage. They also argued that there is a strong correlation between nectar abundance and seed yield. Płażek et al. (2023)^64^ only established that there was a correlation between nectar and seed mass, while any of the sugar contents had no impact on the seed yield. A characteristic feature of common buckwheat is the low percentage of seeds set in relation to the produced inflorescences. According to Jacquemart et al. (2012)^65^ this is due to internal defects, i.e. male or female sterility or abnormalities of the embryo. According to (Słomka et al. 2017)^66^ and Płażek et al. (2023)^64^ there was a very high pollen viability (99%) and germination of all Polish buckwheat varieties tested, so the main reason for the lack of fertilisation was a high percentage of degenerated embryo sacs.

Moreover, Lee and Heimpel (2003)^67^ demonstrated that buckwheat secreted more quantities of nectar in the early morning. In the study by Płążek et al. (2023)^64^ there was no considerable difference in hexose content assessed during two flowering stages, but some differences were noted between the genotypes. The volume and mass of buckwheat nectar depend on the genotype, the time of day (illumination) and the position of the flower on the plant^36,66, 68^. Nectar secreted in the early flowering phase is characterised by greater mass and volume in most buckwheat genotypes. In our study, more nectar was found in the buckwheat crop monoculture. It contained more individual sugars than in the full flowering phase. Buckwheat flowers for a long time during its vegetation period, enabling various insects to frequently visit its inflorescences. Płażek et al. (2023)^64^ showed a positive correlation between total sugars and amino acids in nectar and seed yield in buckwheat varieties. Cawoy et al. (2008; 2009)^35,68^ also led positive correlation between the amount of nectar secretion of buckwheat flowers and the frequency of visits by pollinators. The increase in the level of nectar secretion by the plant attracts a greater number of bees and other insects, increasing the number of visits to the flowers, thus improving the possibility of their cross-pollination. Despite the fact that plants may have a very high number of flowers, only 5–10% of them form seeds^69,70^, which is mostly due to the allogamy of buckwheat^65^. Buckwheat flowers develop early in the morning and bloom only for one day. To pollinate a single flower, at least 10 grains of foreign pollen are necessary, and the time required for this process is less than one hour^71^. In Poland, Germany, Korea, and the US, the main buckwheat pollinators are *Apidae* (honeybee and bumblebee). A worker bee (*Apis mellifera*) visits an average 14–20 flowers/min and works buckwheat for 4–5 h/day, effectively transferring pollen between the plants. Other bees (f. in. from *Syrphidae*) can also make a significant contribution to buckwheat pollination, especially in Asia^65,72^.

The decline in numbers of pollinating insects in agricultural areas has been observed for several decades, partly due to the simplification of the landscape within intensive agro-ecosystems^69^. Intensive agriculture is one of the main causes of recent insect number declines. Such loss of biodiversity has created the need to develop integrated farming methods combining high yields with biodiversity conservation. Intercropping has been shown to lead to a significant increase in bumblebee and honeybee activity, as well as in bee species diversity^25^. Intercropping systems have different types of impacts on pollinators abundance, richness and diversity. In a few cases, they enhance pollinator's abundance and diversity ^17,19^. While in other some cases, intercropping did not alter pollinator's abundance. These phenomenon may depend on the amount of sharing common pollinators among the co-blooming plants^18^.

On farms with perennial cropping systems such as orchards, vineyards or energy crop plantations, long-term ground cover may also be a desirable outcome.

Pollination as an ecosystem service can significantly increase production of more than 70% of agricultural crops grown around the world^73,74^. Pollination of both wild and agricultural plants is carried out by bees and other pollinating insects^75^. Bees are significant in terms of pollination due to their need to feed on pollen and nectar and their use of plants as breeding sites^74^.

AFS constitute an alternative solution for improving environmental conditions in agroecosystems^76^. The suggestion to diversify the area promotes species diversity, increases vegetation layers, increases vegetation shading, reduces harmful effects of high temperatures and improved microclimatic conditions of both the environment and the soil^77^. It our case it was clear that increased shading favoured increased bee abundance in the area. For that reason such a solution would be preferable, also for of long-term climate change mitigation by AFS, as relevant changes also severely affected the behaviour of pollinating bees^75–78^.

## Conclusions

Intercropping buckwheat with paulownia can provide a food source for some insect species on arable land. While the contribution of intercropping per unit area to insect conservation may be small, its potential support for biodiversity could be enormous. Buckwheat has a high beekeeping value and intercropping with paulownia results in an increase in the average number of flowers per plant per day of observation and the average number of flowers in millions per hectare per day of observation. Millions of flowers are present during the buckwheat flowering, maintaining a high density of wild pollinators due to considerable nectar resources. Results from three growing seasons indicate that intercropping buckwheat together with Oxytree did not result in a decrease in the values of parameters relevant to the beekeeping value of buckwheat in terms of its attractiveness to pollinators. Nor did the intercropping of buckwheat between rows of Paulownia trees result in a deterioration of the biometric and yield characteristics of this annual crop. There are currently no data available to compare the results of beekeeping value in an agroforest system consists of paulownia and buckwheat. However, the intercropping of buckwheat together with paulownia has proven to enable efficient use of the area between the rows of Oxytree without deteriorating the conditions for both nectarisation and yield of this crop.

## Data Availability

All data generated or analysed during this study are included in this published article.
